# EVALUATION OF CLINICAL ASPECTS OF PATIENTS UNDERGOING SACRAL NEUROMODULATION FOR FECAL INCONTINENCE: A RETROSPECTIVE MULTICENTER STUDY

**DOI:** 10.1590/S0004-2803.24612025-154

**Published:** 2026-06-05

**Authors:** Hugo Parra de CAMARGO, Vanessa Foresto MACHADO, Rogério Serafim PARRA, Omar FÉRES, José Joaquim Ribeiro da ROCHA, Sthela Maria Murad REGADAS, Luiz Henrique Cury SAAD, Gilmara Pandolfo ZABOT, Luciana Amaral de Retamal MARZÁN, Marcos Antonio DAL PONTE, Aline Landim MANO, Carina Quaquio MESQUITA, Paulo José Pereira de Campos CARVALHO, Marley Ribeiro FEITOSA

**Affiliations:** 1 Universidade de São Paulo, Faculdade de Medicina de Ribeirão Preto, Divisão de Coloproctologia do Departamento de Cirurgia e Anatomia, Ribeirão Preto, SP, Brasil.; 2 Universidade Federal do Ceará, Fortaleza, CE, Brasil.; 3 Faculdade de Medicina de Botucatu, UNESP, Botucatu, SP, Brasil.; 4 Hospital Moinhos de Vento, Serviço de Cirurgia de Cólon e Reto, Porto Alegre, RS, Brasil.; 5 Hospital Federal da Lagoa, Rio de Janeiro, RJ, Brasil.; 6 Universidade de Caxias do Sul, Caxias do Sul, RS, Brasil.; 7 Hospital São Rafael - Rede D`or, Salvador, BA, Brasil.; 8 Centro de controle de disfunções urinárias e intestinais, São Paulo, SP, Brasil.; 9 Hospital Israelita Albert Einstein, Gastroenterologia, São Paulo, SP, Brasil.

**Keywords:** Fecal incontinence, epidemiology, quality of life, Incontinência fecal, epidemiologia, qualidade de vida

## Abstract

**Background:**

Fecal incontinence affects approximately 5%-10% of non-institutionalized individuals and is associated with socioeconomic burden. A meaningful proportion of patients fail conservative management and may require interventional therapies. Sacral neuromodulation has emerged as a less invasive option with favorable outcomes in clinical practice.

**Objective:**

To describe clinical and demographic characteristics and to assess fecal incontinence severity and fecal urgency before and after sacral neuromodulation implantation in Brazilian centers.

**Methods:**

Observational study including patients who underwent sacral neuromodulation implantation for fecal incontinence refractory to conservative treatment. Clinical-demographic variables, previous treatments, surgical implantation aspects, and the Wexner fecal incontinence score before and after the procedure were collected. All patients underwent anorectal manometry. Additional examinations were performed according to clinical indication. The study was approved by the local Ethics Committee.

**Results:**

A total of 87 patients were included; 72 (82.8%) were female, with a mean age of 61.22±16.32 years. Most patients were economically active (56.3%), had completed secondary education (44.8%), and were married (60.9%). The most frequent etiology was neurogenic (42; 48.3%), with a mean disease duration of 73.9±61.33 months. Among women, 17 were nulliparous. Previous surgeries included perineal procedures in 22 (25.3%), colorectal surgery in 16 (18.4%), and pelvic surgery in 12 (13.8%). Only 12 (13.8%) patients had undergone prior surgery specifically for fecal incontinence. Additional tests included endoanal ultrasound (52.9%), colonoscopy (26.4%), electromyography (31%), and pelvic magnetic resonance imaging (2.3%). The median Wexner score decreased after implantation (16; interquartile range: 12-17 vs 2; interquartile range: 0-4), and fecal urgency frequency decreased (51.7% vs 26.4%).

**Conclusion:**

In Brazilian centers, sacral neuromodulation was associated with clinically meaningful reductions in fecal incontinence severity and fecal urgency among patients refractory to conservative treatment, with a safety profile consistent with the literature.

## HIGHLIGHTS

• Multicenter Brazilian real-world cohort (nine reference centers) of 87 adults with fecal incontinence undergoing sacral neuromodulation.

• Sacral neuromodulation produced a marked improvement in fecal incontinence severity (median Wexner 16 [IQR 12-17] to 2 [IQR 0-4]).

• Fecal urgency prevalence decreased substantially after implantation (51.7% to 26.4%), supporting clinically meaningful symptom relief beyond score reduction.

• Low adverse-event rate (6.9%), mainly lead infection (4.6%), consistent with an acceptable safety profile.

## RESUMO

**Contexto:**

A incontinência fecal acomete cerca de 5% a 10% de indivíduos não institucionalizados e está associada a impacto socioeconômico. Uma proporção relevante de pacientes apresenta falha ao tratamento conservador e pode necessitar de intervenção. A neuromodulação sacral surgiu como alternativa menos invasiva, com resultados favoráveis na prática clínica.

**Objetivo:**

Descrever características clínicas e demográficas e avaliar a gravidade da incontinência fecal e a urgência fecal antes e após o implante de neuromodulação sacral em centros brasileiros.

**Métodos:**

Estudo observacional com pacientes submetidos ao implante de neuromodulação sacral por incontinência fecal refratária ao tratamento conservador. Foram coletadas variáveis clínicas e demográficas, histórico de tratamentos prévios, aspectos cirúrgicos do implante e o escore de incontinência fecal de Wexner antes e após o procedimento. Todos os pacientes realizaram manometria anorretal. Exames complementares foram realizados conforme indicação clínica. O estudo foi aprovado pelo Comitê de Ética em Pesquisa da instituição.

**Resultados:**

Foram incluídos 87 pacientes, sendo 72 (82,8%) do sexo feminino, com idade média de 61,22±16,32 anos; 56,3% eram economicamente ativos, 44,8% tinham escolaridade de ensino médio e 60,9% eram casados. A etiologia mais frequente foi neurogênica (42; 48,3%), com tempo médio de doença de 73,9±61,33 meses. Entre as mulheres, 17 eram nulíparas. Cirurgias prévias incluíram procedimentos orificiais em 22 (25,3%), cirurgias colorretais em 16 (18,4%) e cirurgias pélvicas em 12 (13,8%). Apenas 12 (13,8%) pacientes haviam sido submetidos a tratamento cirúrgico prévio especificamente para incontinência fecal. Exames adicionais incluíram ultrassonografia endoanal (52,9%), colonoscopia (26,4%), eletromiografia (31%) e ressonância magnética de pelve (2,3%). Observou-se redução da mediana do escore de Wexner após o implante (16; intervalo interquartil: 12-17 vs 2; intervalo interquartil: 0-4) e redução da frequência de urgência fecal (51,7% vs 26,4%).

**Conclusão:**

Em centros brasileiros, a neuromodulação sacral associou-se a reduções clinicamente relevantes na gravidade da incontinência fecal e na urgência fecal em pacientes refratários ao tratamento conservador, com perfil de segurança consistente com a literatura.

## INTRODUCTION

Fecal incontinence (FI) is the recurrent, involuntary loss of liquid or solid stool and can affect individuals across the lifespan, imposing a substantial psychosocial and functional burden^1^ ^-^ ^3^. Population-based studies estimate a prevalence of 5-10% in the noninstitutionalized population^4^, with higher rates among older adults and individuals with comorbidities. Underreporting is common due to embarrassment, contributing to missed diagnoses and undertreatment^1^ ^,^ ^3^. FI is typically classified as urge, passive, or mixed, and severity is commonly quantified using validated instruments such as the Jorge-Wexner score^1^ ^,^ ^3^.

Evaluation is typically stepwise and includes a detailed clinical history, physical examination, and selective anorectal testing (e.g., anorectal manometry and endoanal ultrasonography) to define underlying mechanisms and guide therapy^2^. First-line management focuses on stool modulation, pelvic floor muscle training with biofeedback, and adjunctive pharmacologic measures. Patients with persistent, moderate-to-severe symptoms despite conservative treatment may be considered for injectable bulking agents or surgical options^2^ ^,^ ^3^ ^,^ ^5^.

Sacral neuromodulation (SNM) has emerged as a minimally invasive option for fecal incontinence refractory to conservative therapy. By modulating sacral pathways, SNM may influence anorectal reflexes, sphincter function, rectal sensation, and colonic motility. Cohort studies and registry data have shown clinically meaningful and durable reductions in incontinence episodes, with acceptable safety profiles^6^ ^-^ ^8^. However, evidence from Latin America remains limited, which restricts external validity and may hinder broader regional implementation^9^.

This multicenter Brazilian study aimed to evaluate the clinical impact of sacral neuromodulation on fecal incontinence severity-assessed by the Wexner score-and on fecal urgency among adults with fecal incontinence after failure of conservative therapy. The findings aim to contextualize real-world outcomes and inform patient selection in our setting.

## METHODS

### Study design and setting

We conducted a retrospective, multicenter cohort study at nine Brazilian reference centers between June 2020 and December 2021: the Division of Coloproctology, Department of Surgery and Anatomy, Ribeirão Preto Medical School, University of São Paulo; Federal University of Ceará (CE); Botucatu Medical School, São Paulo State University (UNESP); Department of Colon and Rectal Surgery, Moinhos de Vento Hospital, Porto Alegre (RS); Lagoa Federal Hospital (RJ); University of Caxias do Sul (RS); São Rafael Hospital, Rede D’Or, Salvador (BA); Urinary and Bowel Dysfunction Control Center (SP); and Hospital Israelita Albert Einstein (SP), all in Brazil. Consecutive adult patients with FI who underwent sacral neuromodulation (SNM) implantation during the study period were identified via the electronic medical record (EMR). In addition to standardized chart abstraction, a brief, study-specific questionnaire-pilot-tested for clarity and administered in Portuguese by trained staff via telephone or clinic visit ≥30 days after definitive (stage-2) implantation-was used to capture patient-reported variables not routinely available in the EMR.

### SNM procedure

All centers used a standardized two-stage technique targeting the S3 foramen under fluoroscopic guidance. Stage 1 involved placement of a tined lead (unilateral, side at surgeon’s discretion). Patients then underwent a 7-14-day test phase; successful response-predefined as ≥50% reduction in FI episodes and/or urgency-prompted stage-2 implantation of the implantable pulse generator (Medtronic^TM^/INTERSTIM II ref 3058). Initial programming parameters were harmonized across centers (14 Hz, 210 μs, amplitude titrated to comfortable sensory response). Perioperative antibiotic prophylaxis followed local protocols.

### Eligibility criteria

Inclusion criteria were age ≥18 years; clinical diagnosis of FI (≥2 episodes/month and Wexner ≥10, and Rome IV criteria); SNM stage-2 implantation performed ≥30 days before data capture; and availability of paired pre- and post-implant outcomes. Patients with obstructed evacuation also reported symptoms of fecal incontinence; obstructed evacuation was identified on anorectal manometry. Exclusion criteria were inadequate records for primary endpoints, loss to follow-up before post-implant assessment, inability to complete the questionnaire, or withdrawal of consent for the survey component. Patients with inflammatory bowel disease, those who had undergone a specific type of surgery and/or were under oncologic follow-up, or post-radiotherapy patients were not excluded.

### Variables and data sources

Trained abstractors used a prespecified codebook to collect data on demographics, clinical characteristics (including FI etiology and duration, urinary incontinence, and obstructed defecation), obstetric history, prior anorectal/colorectal/pelvic surgeries, and ancillary tests (anorectal manometry, endoanal ultrasonography, electromyography, pelvic MRI). Anorectal manometry followed the IAPWG/London Classification protocol. Endoanal ultrasonography and electroneuromyography were performed according to local standards to assess anal sphincter and pelvic floor structure and function. Outcomes included the Wexner score and presence of fecal urgency, evaluated at baseline (≤60 days before implant) and at first post-implant assessment (30-180 days). All identifiers were removed after data linkage to ensure patient privacy.

### Statistical analysis

Continuous variables are presented as median (IQR) or mean±SD, as appropriate. Within-subject pre-post comparisons used the Wilcoxon signed-rank test for Wexner and McNemar’s test (exact when indicated) for urgency. We report effect sizes with 95% CIs (Hodges-Lehmann median difference for Wexner; absolute paired risk difference and discordant-pair OR for urgency). Normality was assessed with the Shapiro-Wilk test. Complete-case analyses were the primary approach; sensitivity analyses used cluster-robust standard errors by center. A two-tailed *P* value <0.05 was considered statistically significant. Analyses were performed using SPSS software, version 20.0 (IBM Corp., Armonk, NY, USA). For the comparison of FI severity scores, as assessed by the Wexner score before and after treatment, the Wilcoxon signed-rank test was used because of the ordinal nature of the data and the lack of an assumption of normality. To evaluate the change in the prevalence of fecal urgency between the pre- and post-treatment assessments (dichotomous variable: presence vs absence), McNemar’s test was applied, as it is appropriate for comparing paired proportions in the same group of patients.

### Ethics

The protocol was approved by the Research Ethics Committee of Hospital das Clínicas, FMRP-USP (CAAE: 31578920.0.0000.5440) on March 15, 2020. 4.029.830. Retrospective chart review was conducted under a waiver of consent; participants provided informed consent for the questionnaire. Data were pseudonymized prior to analysis. The study adheres to the Declaration of Helsinki and follows STROBE recommendations.

## RESULTS

A total of 87 patients who underwent SNM implantation were evaluated. Most were female (72; 82.8%), with a mean age of 61.2±16.3 years. Forty-nine patients (56.3%) were economically active, 39 (44.8%) had completed high school, and 53 (60.9%) were married. The most common FI etiology was neurogenic (42; 48.3%), with a mean symptom duration of 73.9±61.3 months. Urinary incontinence was absent in 61 patients (70.1%), and obstructed defecation symptoms were reported by 16 (18.4%).

Regarding obstetric history among female patients, the mean number of pregnancies was 2.2±2.0, and 55 (76.4%) were multiparous. Vaginal delivery was the most frequent route (46; 63.9%), and forceps were used in 6 deliveries (8.3%). Orificial surgery was the most common non-obstetric procedure (22; 25.3%). Table 1 summarizes the overall clinical characteristics of the sample.

**TABLE 1:** General characteristics of patients included in the study.

Characteristics	Value n(%)
Gender	
**Female**	72 (82.8)
**Age, years (mean± SD)**	61.2±16.3
**Occupation**	
Employed	49 (56.3)
**Education**	
Elementary	11 (12.6)
High school	39 (44.8)
Higher education	37 (42.5)
**Marital status**	
Married	53 (60.9)
Separated	11 (12.6)
Single	12 (13.8)
Widower	11 (12.6)
**Etiology of FI**	
Surgical	12 (13.8)
Idiopathic	9 (10.3)
Neurogenic	42 (48.3)
Obstetric	18 (20.7)
Other	6 (6.9)
**Duration of FI, months**	73.9±61.3
**Urinary incontinence**	
Yes	26 (29.9)
**Obstructed evacuation** n (%)	
Yes	16 (18.4)
**Obstetric history**	
Pregnancies (mean±SD)	2.2±2
Nulliparous- n (%)	17 (23.6)
Multiparous	55 (76.4)
Vaginal birth	46 (63.9)
Abdominal birth	24 (33.3)
Forceps	6 (8.3)
**Surgeries**	
Orificial	22 (25.3)
Colorectal	16 (18.4)
Pelvic	12 (13.8)

SD: standard deviation. FI: fecal incontinence.

Before SNM implantation, 79 patients (90.8%) received conservative treatment, and 12 (13.8%) underwent a prior surgical intervention (Table 2). As part of the pre-implant evaluation, all patients underwent anorectal manometry; 46 (52.9%) underwent endoanal ultrasonography, 27 (31.0%) anorectal electromyography, 23 (26.4%) colonoscopy, and 2 (2.3%) pelvic magnetic resonance imaging (Table 3).

**TABLE 2:** Treatment performed before SNM implantation.

Treatments	n (%)
Clinical management	79 (90.8)
Biofeedback	75 (86.2)
TNS	44 (50.6)
Surgery	12 (13.8)

SNM: sacral neuromodulation.TNS: tibial nerve stimulation.

**TABLE 3:** Additional tests performed before SNM implantation.

Exams	n (%)
Anorectal manometry	87 (100.0)
Endoanal ultrasound	46 (52.9)
Magnetic resonance Imaging	2 (2.3)
Colonoscopy	23 (26.4)
Anorectal electromyography	27 (31.0)

SNM: sacral neuromodulation.

Adverse events related to SNM implantation occurred in 6 patients (6.9%). The most frequent complication was lead infection in 4 patients (4.6%), followed by new-onset urinary incontinence in 2 (2.3%). The mean interval between the first- and second-stage procedures was 16.7±7.1 days.

A reduction in the median Wexner score (16 [IQR 12-17] vs 2 [IQR 0-4]) and in the frequency of fecal urgency (51.7% vs 26.4%) was observed (Table 4). Overall, fecal incontinence severity improved significantly after SNM. The mean Wexner score decreased from 15.0±3.0 at baseline to 2.9±3.4 after treatment (Wilcoxon signed-rank test, W=0, *P*=5.07 × 10^-16^; *P*<0.001).

**TABLE 4:** Characteristics of evacuation function before and after SNM implantation.

Characteristics	Pre - SNM	Post - SNM	*P* **value**
Wexner (median, IQR)	16 (12-17)	2 (0-4)	<0.001
Fecal urgency	45 (51.7)	23 (26.4)	<0.001

SNM: sacral neuromodulation. IQR: interquartile range.

Fecal urgency also decreased substantially, from 45/87 patients (51.7%) at baseline to 23/87 (26.4%) after treatment; this reduction was statistically significant (McNemar test, *P*<0.001).

## DISCUSSION

In this multicenter Brazilian cohort, SNM was associated with a marked reduction in fecal incontinence severity and in the prevalence of fecal urgency. The sample was predominantly female, and neurogenic etiology was the most frequent cause. These real-world data from nine national reference centers support the effectiveness of SNM in patients with fecal incontinence refractory to conservative therapy.

The age distribution and female predominance observed in our cohort mirror well-described epidemiologic patterns of fecal incontinence, including a higher burden among older adults and substantial underreporting in community settings^1^ ^,^ ^3^ ^,^ ^10^ ^,^ ^11^. Although some studies have reported similar prevalence between sexes in specific populations^11^, population-based data from the United States suggest that women are more likely to report fecal incontinence when urinary incontinence coexists^12^). Factors associated with fecal incontinence in older adults-such as lower physical activity, reduced fiber intake, and hormonal status-also align with the profile of our patients^13^.

Unlike series in which obstetric injury predominates, our cohort was enriched for neurogenic etiology (48.3%). In a Latin American multicenter study, obstetric causes accounted for 46% of cases, and most patients reported “significant” improvement after SNM^9^. Reported predictors of success include age <65 years, absence of sphincter injury, and an intraoperative motor and/or sensory response^14^. Differences in etiologic composition and periprocedural selection may influence the magnitude of the observed effect and limit direct comparability across studies.

Accordingly, stratifying outcomes by etiology (neurogenic vs obstetric vs other) and by sphincter integrity, with adjustment for age and parity, would be informative; ideally, effect sizes (e.g., ΔWexner and responder rate) could be estimated and an etiology-by-treatment interaction tested. This approach would strengthen external validity and better position our cohort within the context of the literature.

The decrease in Wexner score observed in this cohort is consistent with-and of comparable magnitude to-that reported in series with long-term follow-up (≥5-10 years)^5^ ^,^ ^15^ ^-^ ^17^. Trial and registry data corroborate the durability of benefit and sustained device use over time, although reprogramming and, occasionally, revision procedures may be required^6^ ^,^ ^7^ ^,^ ^15^. In the present study, the absence of a binary clinical response endpoint (e.g., ≥50% reduction in incontinence episodes) limits direct comparability with these cohorts and should be considered when interpreting the findings.

Adverse events were recorded in 6/87 (6.9%) patients, primarily lead infection (4/87; 4.6%) and urinary incontinence (2/87; 2.3%). Event categorization followed standardized terminology. For urinary incontinence, the event was abstracted from the medical record without distinguishing de novo symptoms from worsening of pre-existing symptoms. These findings are consistent with published series, which report infection, lead migration, generator pocket pain, and the need for revision or explantation at frequencies that vary by device and center^6^ ^,^ ^7^ ^,^ ^9^. Taken together, our results suggest a safety profile for SNM that aligns with international experience.

Consistent with guidelines for benign anorectal disorders, these findings support SNM as a second-line option after failure of conservative therapy (dietary measures, pharmacotherapy, and pelvic floor physiotherapy with biofeedback)^2^ ^,^ ^5^. The concomitant improvement in fecal urgency, in addition to the reduction in Wexner score, is clinically meaningful, as urgency is a key determinant of disability in daily activities. These results may inform preoperative counseling regarding realistic expectations, particularly in patients with neurogenic fecal incontinence.

Key strengths of this study include its multicenter design, the real-world clinical sample, and the use of a standardized outcome measure (Wexner score), together with a regionally focused scope that remains underreported. These features enhance both generalizability and local relevance. Limitations include the retrospective design, the absence of test-phase data, the lack of binary clinical response and quality-of-life endpoints, potential misclassification of adverse events, and limited characterization of sphincter lesions and imaging findings, which restrict subgroup analyses and comparability with the literature^17^. Overall, these findings provide pragmatic evidence from Brazilian centers while highlighting clear priorities for prospective validation.

Future national studies, ideally prospective, should incorporate: (a) a binary primary endpoint (e.g., ≥50% responder rate); (b) the Fecal Incontinence Quality of Life (FIQL) instrument and other quality-of-life measures; (c) symptom diaries to capture incontinence episodes; (d) systematic documentation of programming parameters and reinterventions; and (e) stratified analyses by etiology and sphincter integrity to refine patient selection.

Taken together, these multicenter real-world data indicate that SNM provides clinically meaningful reductions in symptom burden, with an acceptable safety profile, among patients who have failed conservative therapy. Although interpretation is limited by the retrospective design and the absence of test-phase and quality-of-life endpoints, the magnitude and direction of effect are consistent with long-term series and registry data. As access to SNM expands in Brazil and Latin America, standardized collection of responder rates, episode diaries, quality-of-life measures, and device programming and reintervention data will be essential to refine candidate selection, benchmark outcomes across centers, and support shared decision-making. These considerations contextualize our findings and lead to the conclusion presented below.

## CONCLUSION

In Brazilian centers, SNM was associated with clinically meaningful reductions in FI severity (Wexner score) and in fecal urgency among patients who had failed conservative therapy, with a safety profile consistent with published experience. Interpretation should account for selection conditioned on test-phase success and the absence of diary-based and quality-of-life endpoints. Prospective studies with standardized endpoints are needed to strengthen generalizability and optimize candidate selection.

## Data Availability

Data-available-upon-request
